# Mesenchymal stem cell-derived extracellular vesicles attenuate influenza virus-induced acute lung injury in a pig model

**DOI:** 10.1186/s13287-018-0774-8

**Published:** 2018-01-29

**Authors:** Mahesh Khatri, Levi Arthur Richardson, Tea Meulia

**Affiliations:** 1 Food Animal Health Research Program, Ohio Agricultural Research and Development Center, The Ohio State University, 1680 Madison Avenue, Wooster, OH 44691 USA; 2 Molecular and Cellular Imaging Center, Ohio Agricultural Research and Development Center, The Ohio State University, Wooster, OH USA

**Keywords:** Mesenchymal stem cells, Extracellular vesicles, Influenza, Acute lung injury, Stem cell therapy, Large animal model

## Abstract

**Background:**

Mesenchymal stem (stromal) cells (MSCs) mediate their immunoregulatory and tissue repair functions by secreting paracrine factors, including extracellular vesicles (EVs). In several animal models of human diseases, MSC-EVs mimic the beneficial effects of MSCs. Influenza viruses cause annual outbreaks of acute respiratory illness resulting in significant mortality and morbidity. Influenza viruses constantly evolve, thus generating drug-resistant strains and rendering current vaccines less effective against the newly generated strains. Therefore, new therapies that can control virus replication and the inflammatory response of the host are needed. The objective of this study was to examine if MSC-EV treatment can attenuate influenza virus-induced acute lung injury in a preclinical model.

**Methods:**

We isolated EVs from swine bone marrow-derived MSCs. Morphology of MSC-EVs was determined by electron microscopy and expression of mesenchymal markers was examined by flow cytometry. Next, we examined the anti-influenza activity of MSC-EVs in vitro in lung epithelial cells and anti-viral and immunomodulatory properties in vivo in a pig model of influenza virus.

**Results:**

MSC-EVs were isolated from MSC-conditioned medium by ultracentrifugation. MSC-EVs were round-shaped and, similarly to MSCs, expressed mesenchymal markers and lacked the expression of swine leukocyte antigens I and II. Incubation of PKH-26-labeled EVs with lung epithelial cells revealed that MSC-EVs incorporated into the epithelial cells. Next, we examined the anti-influenza and anti-inflammatory properties of MSC-EVs. MSC-EVs inhibited the hemagglutination activity of avian, swine, and human influenza viruses at concentrations of 1.25–5 μg/ml. MSC-EVs inhibited influenza virus replication and virus-induced apoptosis in lung epithelial cells. The anti-influenza activity of MSC-EVs was due to transfer of RNAs from EVs to epithelial cells since pre-incubation of MSC-EVs with RNase enzyme abrogated the anti-influenza activity of MSC-EVs. In a pig model of influenza virus, intratracheal administration of MSC-EVs 12 h after influenza virus infection significantly reduced virus shedding in the nasal swabs, influenza virus replication in the lungs, and virus-induced production of proinflammatory cytokines in the lungs of influenza-infected pigs. The histopathological findings revealed that MSC-EVs alleviated influenza virus-induced lung lesions in pigs.

**Conclusions:**

Our data demonstrated in a relevant preclinical large animal model of influenza virus that MSC-EVs possessed anti-influenza and anti-inflammatory properties and that EVs may be used as cell-free therapy for influenza in humans.

## Background

Influenza A viruses (IAV) cause an acute respiratory disease in humans and animals. Annual outbreaks and occasional pandemics of influenza result in millions of deaths, suffering, and economic losses. In the US alone, since 2010 influenza viruses have caused 140,000–710,000 hospitalizations resulting in 12,000–56,000 deaths annually (https://www.cdc.gov/flu/about/disease/burden.htm). The elderly, infants, and people with underlying conditions are at high risk of influenza-associated mortality. In addition to seasonal and pandemic viruses, highly pathogenic avian influenza (HPAI) H5N1 virus has been repeatedly transmitted directly from avian species to humans. In humans, H5N1 virus is associated with severe disease resulting in multi-organ failure and high mortality rates [1, 2]. As of 30 October 2017, HPAI H5N1 viruses have caused 860 human infections resulting in 454 deaths since 2003 (http://www.who.int/influenza/human_animal_interface/2017_10_30_tableH5N1.pdf?ua = 1) Severe cases of influenza cause significant mortality due to their ability to induce cytokine-mediated immune lung pathology with features of moderate to severe acute respiratory distress syndrome (ARDS) [3].

Influenza virus infections are generally controlled by annual vaccination. However, these vaccines provide limited protection against new reassortants which are genetically different from the vaccine virus. In the event of a pandemic, generation of a new vaccine containing circulating viruses takes approximately 6 months. Moreover, influenza viruses continually undergo mutations resulting in the generation of new viral strains that can become resistant to currently available antiviral drugs. Thus, alternative therapies capable of inhibiting influenza virus replication and attenuating the inflammatory response of the host are needed.

Mesenchymal stem (stromal) cells (MSCs) are multipotent cells that were first identified in bone marrow (BM) as plastic adherent fibroblast-like cells. MSCs possess multilineage differentiation, and immunomodulatory and tissue repair properties [4]. Due to these properties MSCs are attractive as cellular therapy for inflammatory and autoimmune diseases, and regenerative medicine. Several studies of ARDS in animal models have shown beneficial effects of MSC administration, and clinical trials have shown the feasibility of MSC administration in patients with ARDS [5–7]. Therapeutic studies are underway. Similarly to ARDS, severe influenza virus infections in humans and animal models show acute inflammatory response and lung damage [8]. As MSCs suppress inflammation and have tissue repair and regenerative ability, ARDS and influenza are appropriate targets for MSC therapy. However, MSC therapy in mice models of influenza show inconsistent results [9–12]. Also, we and others have shown that influenza virus infects MSCs and that infection may alter the immunoregulatory and differentiation properties of MSCs [13–15].

Several studies indicated that the beneficial actions of MSCs are due to release of paracrine factors since only a few transplanted MSCs engraft at the site of injury [16]. Recently, extracellular vesicles (EVs) that include exosomes (Exo) which are released from multivesicular bodies and microvesicles (MVs) that are shed from the cell surface, and apoptotic bodies were identified in MSC secretions [17]. In this paper EVs, Exo, and MVs will be collectively referred to as EVs. MSC-derived EVs have similar expression of surface molecules and contain MSC-specific proteins, mRNAs, microRNAs (miRNAs), organelles, and lipids [18–20]. In diseased tissue, MSC-EVs interact with injured cells and transfer proteins, mRNA, and bioactive lipids from MSCs to injured cells resulting in tissue repair [21, 22]. In rodent models, these EVs were as therapeutically efficacious as MSCs in *E. coli* endotoxin-induced acute lung injury (ALI) and *E. coli*-induced severe pneumonia in mice [20, 23].

Due to their close similarity in anatomy, physiology, and immunology to humans, pigs are used as a large animal preclinical model for several human diseases, including respiratory diseases and regenerative medicine [24, 25]. In addition, pigs are naturally infected with influenza virus as the respiratory epithelium of pigs expresses receptors utilized by avian and mammalian influenza viruses [26]. Influenza virus pathogenesis and clinical signs in pigs are also similar to those observed in humans. Thus, pigs are a suitable large animal model to study human influenza virus pathogenesis and to test the efficacy of therapeutics including MSCs or their derivatives for influenza.

## Methods

### Isolation of MSCs and MSC-derived EVs

MSCs from femur bones of 2- to 6-week-old commercial pigs were isolated as described previously [27, 28]. Briefly, the tip of each bone was removed and the marrow was harvested by inserting a syringe needle into one end of the bone and flushing with Dulbecco’s modified Eagle’s medium (DMEM; Gibco). The BM cells were filtered through a 70-μm nylon mesh filter (BD, Falcon, USA) and mononuclear cells were obtained by density gradient centrifugation over Ficoll-Hypaque. Cells (1–5 × 10^5^/cm^2^) were plated in 75-cm^2^ cell culture flasks in DMEM containing 10% fetal bovine serum (FBS; Gibco) and 1% antibiotic solution (Gibco) (C-DMEM). Cultures were incubated at 37 °C in a humidified atmosphere containing 95% air and 5% CO_2_. The nonadherent cells were removed after 72 h of culture and cells were passaged when they were 90% confluent by treating them with 0.25% trypsin containing 0.02% EDTA. MSCs passaged between three and five times were used for the generation of MSC-EVs for in vitro and in vivo experiments. For the isolation of MSC-EVs, MSCs were cultured in C-DMEM in T225 flasks and MSC-EVs were isolated as previously described [23, 29, 30]. Briefly, cells when 80% confluent were washed with serum-free DMEM and cultured in DMEM containing 0.5% bovine serum albumin. After 48 h, conditioned medium (CM) from MSC cultures was collected and centrifuged at 3000 rpm for 20 min to remove the cellular debris. Next, CM was ultracentrifuged at 25,000 rpm for 70 min at 4 °C. The EV pellet was washed with DMEM by ultracentrifugation at 25,000 rpm for 70 min at 4 °C. The EV pellet was then suspended in phosphate-buffered saline (PBS; 10 μl PBS/million MSCs) and further dilutions were made in serum-free DMEM. The protein content of EVs was determined by micro-bicinchoninic acid protein assay kit (Thermofisher Scientific). MSC-EV RNA was isolated using the RNAeasy kit (Qiagen) and RNA concentration was determined by NanoDrop (Thermofisher Scientific).

### Transmission electron microscopy (TEM)

EVs (10 μl) were applied to a formvar/carbon-coated grid for 5 min; after blotting, the grid was stained with 2% aqueous uranyl acetate for 1 min. After blotting, the grids were air-dried and examined under TEM (HITACHI, H-7500, Japan).

### Flow cytometry

MSCs and EVs were examined for surface expression of mesenchymal markers and swine leukocyte antigen (SLA)-I and SLA-II, and EVs were also examined for EV markers (CD9, CD63, and CD81) by flow cytometry as described previously [31]. EVs (30 μg in 50 μl PBS) were incubated with 10 μl of 4-μm diameter aldehyde/sulfate latex beads for 15 min at room temperature followed by the addition of 1 ml PBS and incubation was further continued for 2 h with gentle shaking. The reaction was stopped by incubation for 30 min in 100 mM glycine.

MSCs were detached by treatment with 0.25% trypsin-EDTA and single cell suspensions of MSC and EV-coated beads were stained with the following primary antibodies: mouse anti-pig CD29, mouse anti-human CD90 (BD Biosciences), mouse anti-pig CD44 and SLA-I and SLA-II (VMRD). For the detection of EV markers, EV-coated beads were stained with the following primary antibodies cross-reactive with pig: mouse anti-human CD9 (GeneTex), mouse anti-human CD63 (BD Biosciences), and mouse anti-human CD81 (BD Biosciences) for 20 min at 4 ° C in dark. Cells and beads were washed three times and incubated with secondary antibodies conjugated with allophycocyanin or phycoerythrin for 30 min. Appropriate isotype and secondary antibodies were used as controls for nonspecific binding. Cells and beads were acquired by C6 flow cytometer (BD Accuri Cytometers) and analyzed using CFlow® plus Software (Accuri) as described previously [32].

### Incorporation of EVs in pig lung epithelial cells

We examined whether MSC-EVs had the ability to enter cells using pig lung epithelial cells (MK1-OSU; LECs). MK1-OSU is a spontaneously immortalized cell line established in our laboratory. This cell line was derived from the distal trachea and proximal lung tissue of a 5- to 6-week-old pig. These cells express α2-3- and α2-6-linked sialic acids (receptors for avian and mammalian influenza viruses, respectively), and support the replication of swine, avian, and human-origin influenza viruses (unpublished results). MSC-EVs were labeled using PKH-26 Red Fluorescent Cell Linker Kit (Sigma-Aldrich) as per the manufacturer's instructions. LECs cultured overnight in a 24-well plate were incubated with labeled MSC-EVs for 24 h at 37 °C. To confirm the internalization of EVs inside the cells, the cell cytoplasm was stained using polyclonal rabbit anti-human β-tubulin as the primary antibody (Thermofisher Scientific) and Alexa 488-conjugated goat anti-rabbit secondary antibody (Thermofisher Scientific). Cell nuclei were stained with 4′,6-diamidino-2-phenylindole (DAPI; Thermofisher Scientific Life Sciences). The cells were examined under a fluorescence microscope (Olympus, Japan). For flow cytometry, a single cell suspension was obtained by treating the cells with 0.25% trypsin-EDTA and washing with PBS. Cells were acquired by C6 flow cytometer (BD Accuri Cytometers) and incorporation of PKH-26 labeled EVs in cells was analyzed by CFlow® plus Software (Accuri).

### Hemagglutination inhibition assay

The effect of MSC-EVs on the ability of influenza virus to hemagglutinate red blood cells was examined by hemagglutination inhibition assay. Two-fold dilutions of MSC-EVs (starting from 10 μg/ml) in 25 μl volume were mixed with 8 hemagglutination (HA) units of swine (swine/TX/98; H3N2 and Swine/MN/08; H1N1), avian (Gull/MD/1995; H9N5 and Chicken/NY/H7N2), and human (Human/CA/09; H1N1) influenza viruses and incubated for 20 min at 37 °C followed by the addition of 50 μl 1% turkey red blood cell suspension. Plates were incubated for 30 min at room temperature and the concentration of MSC-EVs capable of inhibiting HA activity of different influenza viruses was determined.

### Effect of MSC-EVs on influenza virus replication and apoptosis in LECs

To examine the effect of MSC-EVs on influenza virus replication, two sets of experiments were conducted. In the first set, we incubated swine/MN/08; H1N1 (SwIV) equivalent to MOI 1 with 10 μg/ml MSC-EVs for 20 min at room temperature. LECs cultured overnight were then infected with SwIV alone or virus-MSC-EV mixture for 1 h at 37 °C. Cells were washed with PBS and were incubated with or without MSC-EVs. After 8 h, influenza virus nucleoprotein (NP) was detected using anti-NP monoclonal antibody in an immunofluorescence assay (IFA). Cells expressing NP protein were counted in at least five microscopic fields and virus titers in culture supernatants were determined by titration in MDCK cells [8]. At 24 h after infection, apoptotic cells were detected by TUNEL assay using ApopTag Fluorescein Apoptosis Detection Kit (EMD Millipore). Apoptotic cells were counted in five microscopic fields and data are expressed as mean ± SD number of apoptotic cells/microscopic field.

In the second set of experiment, LECs were infected with SwIV at an MOI 1 for 1 h at 37 °C, followed by washing with PBS, and LECs were incubated with or without MSC-EVs for 8 h. Influenza-infected cells were detected by the expression of NP by IFA. In some experiments, MSC-EVs were treated with 1 U/ml RNase for 1 h at 37 °C, 1 U/ml RNase was then added to stop the reaction and MSC-EVs were washed by ultracentrifugation [29].

### Immunofluorescence assay

LECs were washed with PBS and fixed with 80% acetone for 10 min at –20 °C. The expression of influenza virus NP in infected cells was detected using mouse anti-NP antibody as a primary antibody and Alexa 488-conjugated goat anti-mouse IgG as a secondary antibody. Nuclei were stained with DAPI (Thermofisher Scientific Life Sciences).

### Animals

Five-week-old conventional large White-Duroc crossbred pigs were obtained from the OSU herd. Maintenance of pigs and all experimental procedures were conducted in accordance with the guidelines of the Institutional Laboratory Animal Care and Use Committee, The Ohio State University (protocol #2014A00000040).

### Experimental design and sample collection

At 8 weeks of age, nine pigs (mean weight 12.1 ± 1.3 kg) were divided into three groups of three pigs each. Pigs in group 1 were inoculated intranasally with DMEM and these pigs served as controls. Pigs in groups 2 and 3 were similarly inoculated with SwIV (5 × 10^6^ TCID_50_ per pig). Pigs had a hemagglutination inhibition (HI) antibody titer of 1:12 ± 4 against SwIV before infection. In our previous study, infection of 8-week-old commercial pigs with SwIV (5 × 10^6^ TCID_50_ per pig) induced extensive lung lesions [33]. Twelve hours after SwIV infection, pigs in groups 2 and 3 were administered intratracheally with DMEM and MSC-EVs (80 μg/kg body weight (BW)), respectively. In human clinical trials for ARDS [6], MSCs between 1 and 10 × 10^6^ cells/kg BW were used. In our study, EV dose per kg BW was calculated by the protein content of EVs produced by 10 × 10^6^ MSCs cultured for 48 h. In a previous study [23] intratracheally administered EVs were effective in treating *E. coli*-induced severe pneumonia. Based on this observation, in this study EVs were administered by the intratracheal route. Pigs were monitored daily for clinical signs and nasal swabs were collected at 1 and 3 days post-infection (DPI) for virus titration in MDCK cells. At DPI 3, pigs were euthanized and lungs were harvested. Lung lysate was prepared from the left lung for virus quantification and cytokine analysis, and the entire right lung was fixed in 10% buffered formalin for microscopic examination. The time-point DPI 3 was selected based on our earlier publication showing higher virus replication and proinflammatory cytokine production at DPI 3 compared with DPI 6 [8]. Paraffin-embedded sections of lung tissues were stained with hematoxylin and eosin and examined with the help of a trained pathologist who was not aware of the experimental design. The slides were examined for bronchiolar epithelial changes, peribronchiolar inflammation, and interstitial pneumonia, and lesions were scored from 0–3 as described previously [34, 35].

### Detection of SwIV in nasal swabs and lungs

A 10% (w/v) lung homogenate was prepared from left apical lobe lung tissue and virus titer was determined by titration in MDCK cells as described [33]. Virus titers were calculated by the Reed and Muench method.

### Detection of cytokines in lungs

Lung lysates from pigs were prepared and levels of tumor necrosis factor (TNF)α, CXCL10, and interleukin (IL)-10 in lung lysates were determined by enzyme-linked immunosorbent assay (ELISA) as described previously [8].

### Statistical analysis

In vitro data on MSC-EV-mediated inhibition of SwIV replication were analyzed by Student’s *t* test. Microscopic lung lesions, virus titers, and cytokine concentrations between groups of pigs were compared using Kruskal-Wallis test. *P* values < 0.05 were considered statistically significant.

## Results

### Characteristics of MSC-derived EVs

**Fig. 1:**
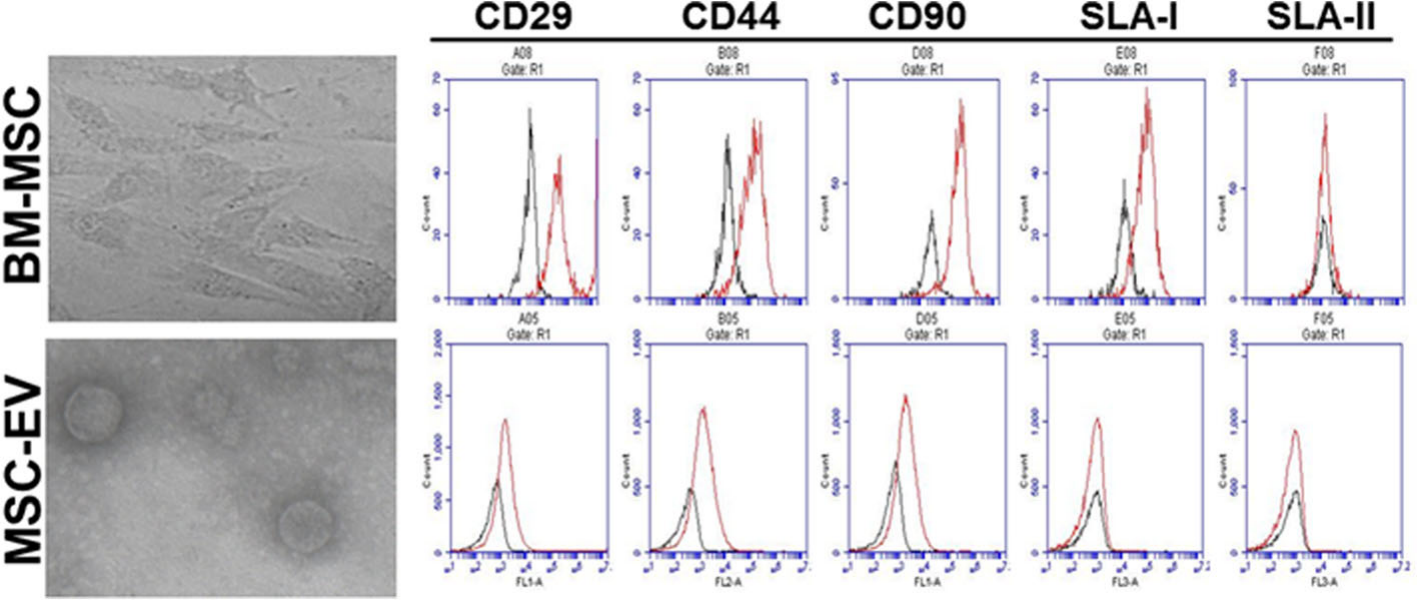
Characteristics of MSC-derived EVs. Extracellular vesicles (EVs) were isolated from the conditioned medium of porcine bone marrow-derived mesenchymal stem cells (BM-MSCs) by ultracentrifugation. Morphology and size of MSC-EVs was examined by TEM. EVs were round-shaped and approximately 100 nm in size (×25 K). Expression of mesenchymal markers on MSCs and MSC-EVs was examined by flow cytometry. MSCs expressed the mesenchymal markers CD29, CD44, and CD90, and swine leukocyte antigen (SLA)-I, but SLA-II was not expressed. Similarly to MSCs, EVs expressed the mesenchymal markers but lacked the expression of SLA-I and SLA-II (black line: isotype staining; red line: specific staining)

**Fig. 2:**
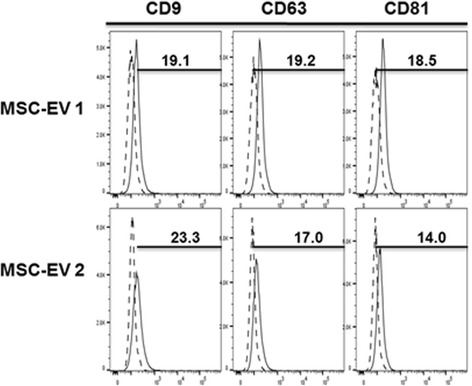
Mesenchymal stem cell extracellular vesicles (MSC-EVs) express EV markers. EV-coated latex beads were examined for the expression of EV markers by flow cytometry. EVs expressed the specific EV markers CD9, CD63, and CD81 (broken line: isotype staining; solid line: specific staining)

We isolated MSCs from the BM of 2- to 6-week-old pigs that showed characteristic features of MSCs, such as adherence to a plastic surface, fibroblast-like morphology (Fig. 1), self-renewal potential, and high in vitro proliferation capacity and differentiation potential (data not shown). Colony-expanded MSCs showed the expression of the mesenchymal markers CD29, CD44, CD90, and SLA-I, but SLA-II was not detected on these cells. MSC-EVs were round-shaped and, similarly to MSCs, EVs isolated from BM-MSCs also expressed mesenchymal markers; however, unlike MSCs, they lacked the expression of both SLA-I and II (Fig. 1). MSC-EVs also expressed EV-specific markers such as CD9, CD63, and CD81 (Fig. 2). We also determined the RNA and protein concentration in MSC-EVs. MSC-EVs contained 113 ± 37 ng/100 μl EVs (*n* = 5) total RNA and 79 ± 1 μg/100 μl EVs (*n* = 4) total protein.

**Fig. 3:**
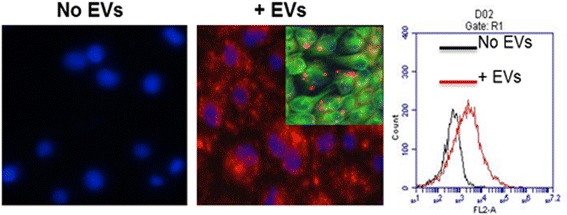
MSC-EVs incorporate into lung epithelial cells. PKH-26 labeled extracellular vesicles (EVs) were incubated with LECs for 24 h. Incorporation of EVs in LECs was examined by fluorescent microscope and flow cytometry. The inset in the middle panel shows the internalization of EVs in the cytoplasm of LECs. Cellular cytoplasm was stained using β-tubulin antibody (green); EVs (red) were found to be localized inside the LECs (×200)

Additionally, MSC-EVs showed the ability to incorporate into LECs. EVs stained red with PKH-26 dye were found inside the cytoplasm of cells when examined under a fluorescent microscope. Incorporation of MSC-EVs in LECs was also confirmed by flow cytometry (Fig. 3).

### MSC-EVs inhibit the HA activity of influenza viruses

To determine if MSC-EVs possess anti-influenza activity, we first examined if EVs inhibited the HA activity of influenza viruses. Eight HA units of swine/TX/98; H3N2, swine/MN/08; H1N1, gull/MD/1995; H9N5, chicken/NY/H7N2, and human/CA/09; H1N1 influenza viruses were incubated with different concentrations of MSC-EVs and the HA activity of these viruses was examined. MSC-EV concentrations between 1.25 and 5 μg/ml completely inhibited the HA activity of these viruses (Table 1).

**Table 1:** MSC-EVs inhibit hemagglutination (HA) activity of influenza viruses

Influenza virus	Hemagglutination at EV concentrations (μg/ml)				
	10	5	2.5	1.25	0.625
Swine/TX/1998; H3N2	–	–	+	+	+
Human/CA/09; H1N1	–	–	–	–	+
Swine/MN/08; H1N1	–	–	+	+	+
Gull/MD/1995; H9N5	–	–	+	+	+
Chicken/NY/1995; H7N2	–	–	–	–	+

Eight HA units of swine, human, and avian origin influenza viruses were incubated with different concentrations of mesenchymal stem cell extracellular vesicles (MSC-EVs) or medium control at room temperature for 20 min; 50 μl 1% turkey red blood cells were then added to the wells and MSC-EV-mediated inhibition of HA by influenza viruses was examined. Results are representative of three different experiments using EVs derived from MSCs isolated from three different pigs

### MSC-EVs inhibit SwIV replication and virus-induced apoptosis in LECs

**Fig. 4:**
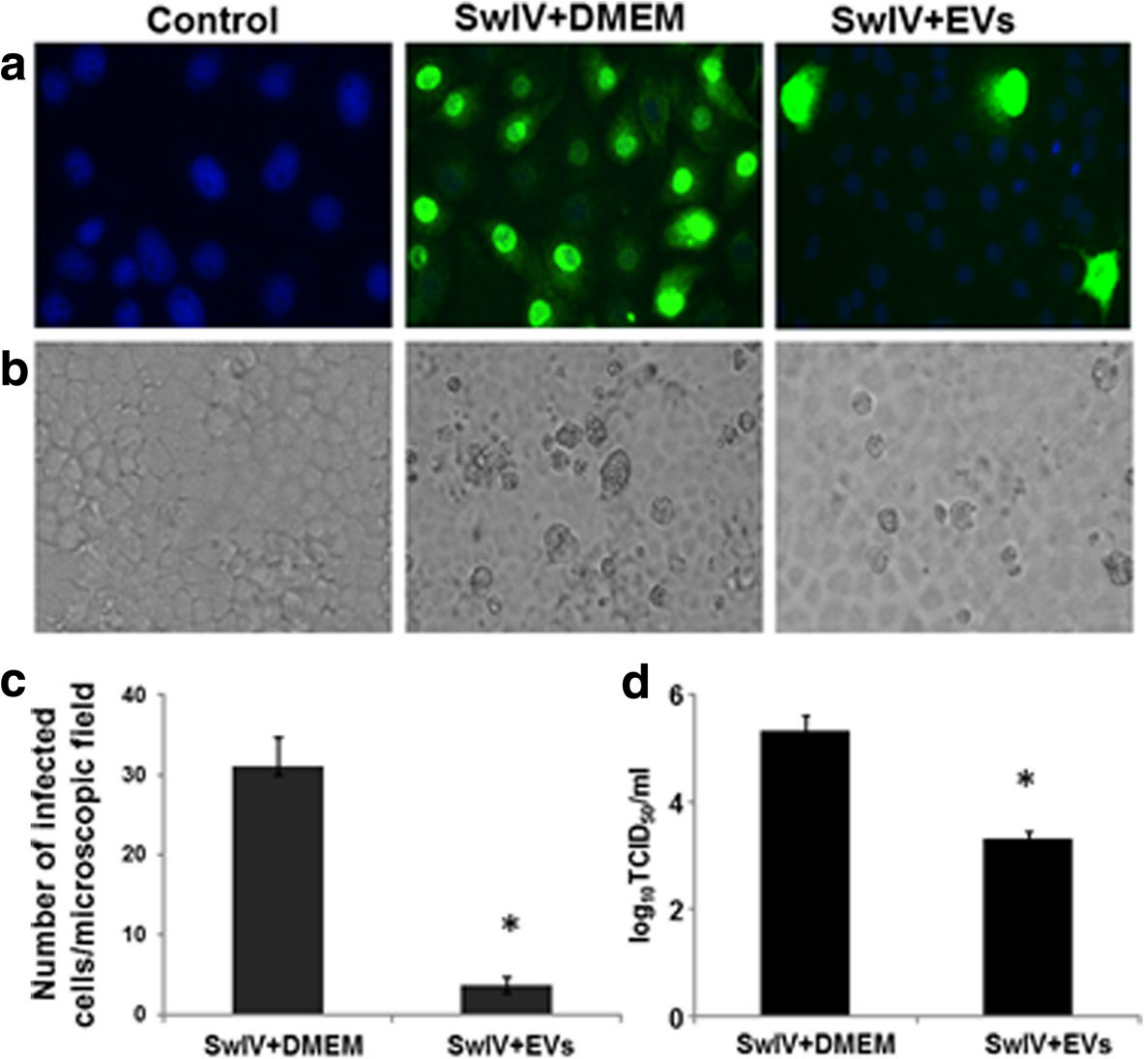
MSC-EVs inhibit influenza virus replication in lung epithelial cells. Swine/MN/08; H1N1 (SwIV; MOI = 1) was incubated with Dulbecco’s modified Eagle’s medium (DMEM) or 10 μg/ml MSC extracellular vesicles (EVs) for 20 min at room temperature. After the incubation, LECs were inoculated with virus-EV mixture or virus alone and incubated for 1 h at 37 °C. Influenza virus NP was detected at 8 h after infection (**a**) and SwIV-induced cytopathology was observed at 48 h after infection (**b**). SwIV-infected cells expressing NP were counted at 8 h after infection. Each bar represents mean ± SD of virus-infected cells in five microscopic fields (20×) (**c**). Virus titers in supernatants of SwIV-infected and MSC-EV-treated cells at 48 h after infection were determined by titration in MDCK cells. Data are expressed as mean ± SD from three independent experiments using EVs derived from BM-MSCs from three different pigs (**d**). *P < 0.05

**Fig. 5:**
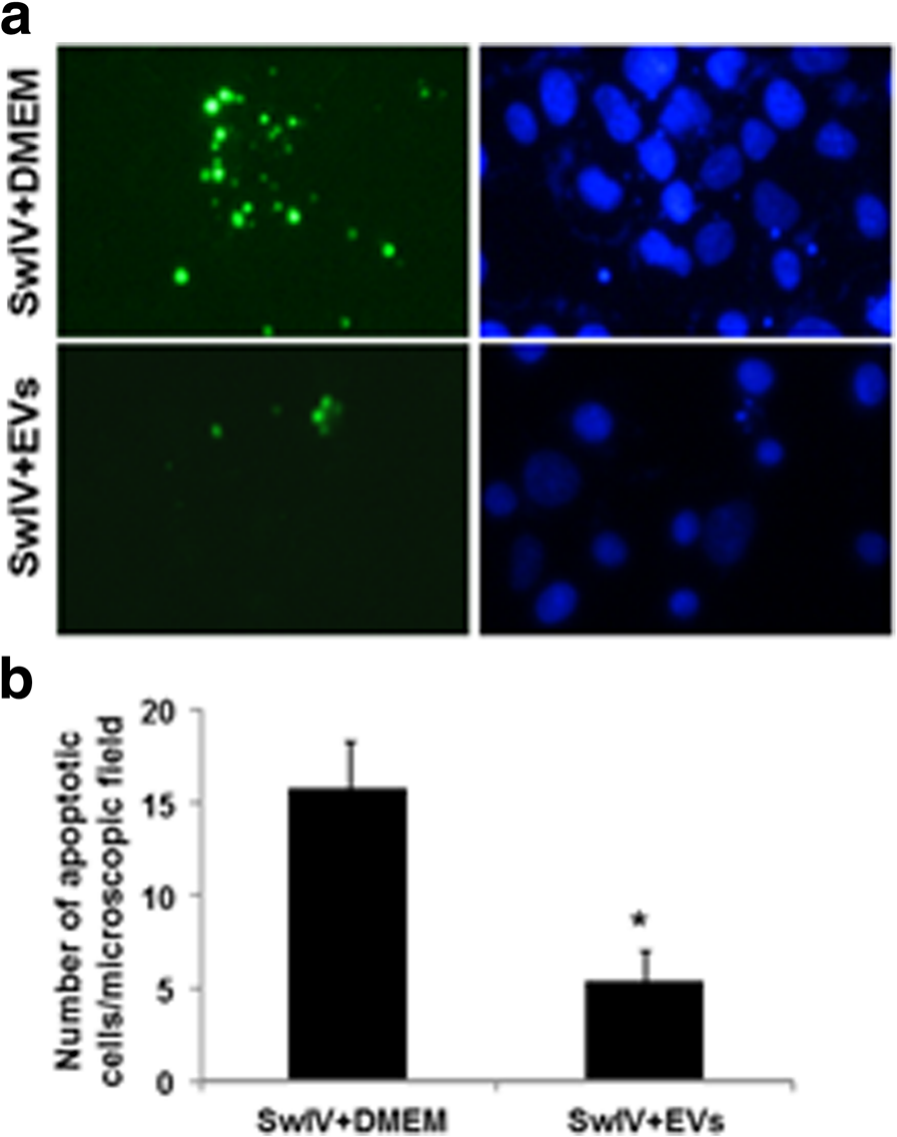
MSC-EVs inhibit influenza virus-induced apoptosis. **a** Swine/MN/08; H1N1 (SwIV; MOI = 1) was incubated with Dulbecco’s modified Eagle’s medium (DMEM) or 10 μg/ml MSC extracellular vesicles (EVs) for 20 min at room temperature. After the incubation, pig lung epithelial cells were incubated for 1 h with SwIV or SwIV pre-incubated with MSC-EVs. At 24 h after infection, apoptotic cells were detected by TUNEL assay using the ApopTag Fluorescein Apoptosis Detection Kit (EMD Millipore). **b** TUNEL-positive cells in SwIV-infected and MSC-EV-treated LECs were counted at 24 h after infection. Values are expressed as mean ± SD of apoptotic cells in five microscopic fields (20×). *P < 0.05

We examined MSC-EVs derived from BM-MSCs for anti-influenza activity. SwIV (Sw/MN/08; MOI of 1) was incubated with MSC-EVs (10 μg/ml) for 20 min at room temperature. LECs were infected with EV-SwIV mixture or virus only. At 8 h after infection, IAV-NP was detected by IFA (Fig. 4a and c). Significantly reduced (*P* < 0.05) influenza replication was detected when cells were infected with SwIV pretreated with MSC-EVs. Consistent with reduced influenza virus replication in LECs treated with MSC-EVs, MSC-EVs also significantly (*P* < 0.05) inhibited the apoptosis of influenza-infected LEC (Fig. 5a and b).

**Fig. 6:**
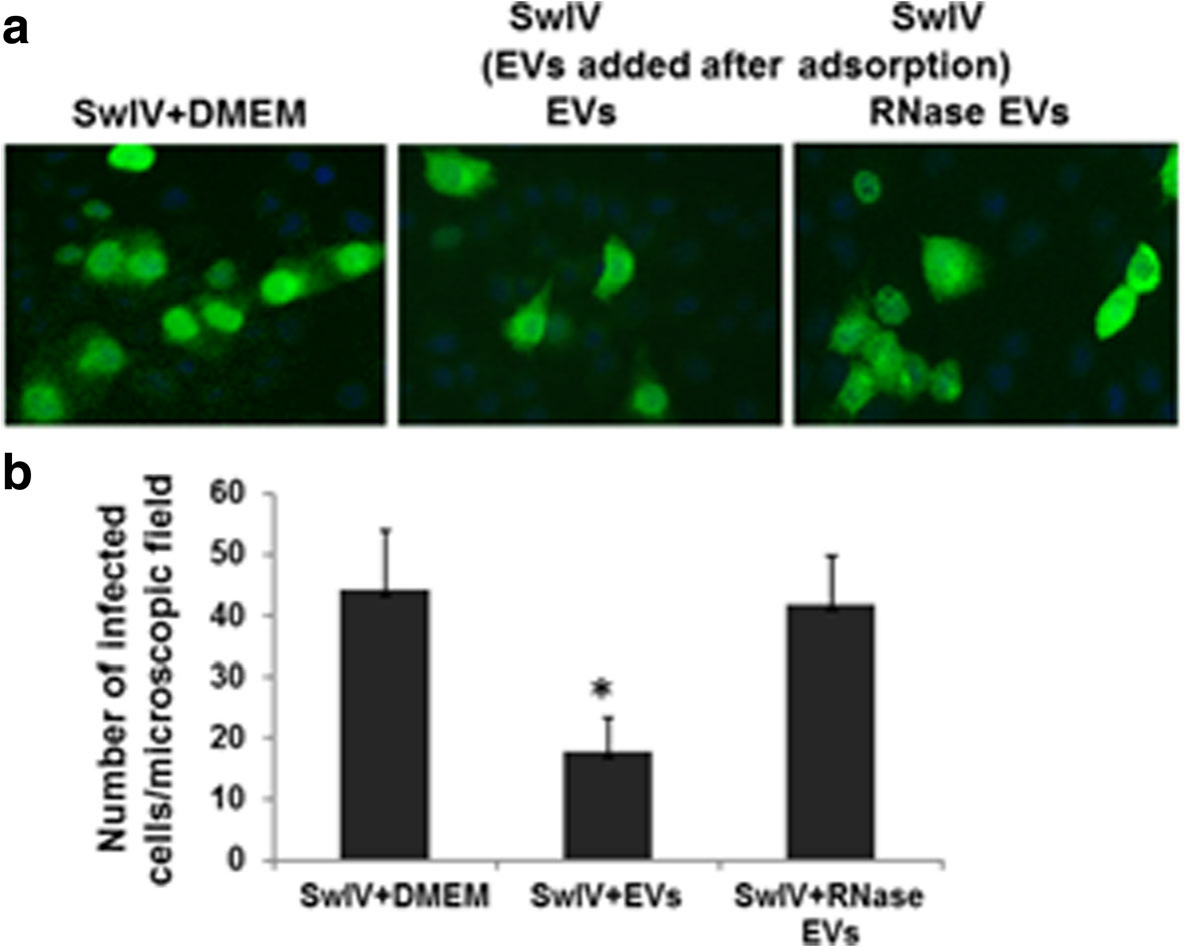
MSC-EVs inhibit influenza virus replication after virus entry in lung epithelial cells. **a** Pig lung epithelial cells were infected with swine/MN/08; H1N1 (SwIV; MOI = 1) for 1 h; after the adsorption, cells were washed and cultured in media only or media containing 10 μg/ml MSC extracellular vesicles (EVs) or RNase-treated MSC-EVs. Influenza virus NP was detected 8 h after infection. **b** Each bar represents mean ± SD of virus-infected cells in five microscopic fields (20×). Experiments were repeated three times using EVs derived from BM-MSCs from three different pigs. *P < 0.05. DMEM Dulbecco’s modified Eagle’s medium

In another set of experiments, porcine lung epithelial LECs were infected with SwIV for 1 h, washed, and incubated with media without or with MSC-EVs (10 μg/ml). At 8 h after infection, IAV-NP was detected by IFA. The addition of MSC-EVs after virus entry significantly (*P* < 0.05) reduced influenza replication in LECs (Fig. 6).

### MSC-EVs attenuate SwIV-induced ALI in pigs

We examined the anti-influenza and immunomodulatory effect of MSC-EVs in influenza virus-induced ALI in pigs. Eight-week-old pigs were infected intranasally with 5 × 10^6^ TCID_50_ of SwIV. Twelve hours after SwIV inoculation, DMEM or MSC-EVs (80 μg/kg) were administered intratracheally in influenza-infected pigs. At days 1 and 3 after EV administration, we collected the nasal swabs, and at day 3 after EV administration the pigs were euthanized and bronchoalveolar lavage (BAL) and lungs were collected. Lungs were homogenized to make 10% lung lysate for determining virus titers, and cytokines were examined in the lung lysate.

**Fig. 7:**
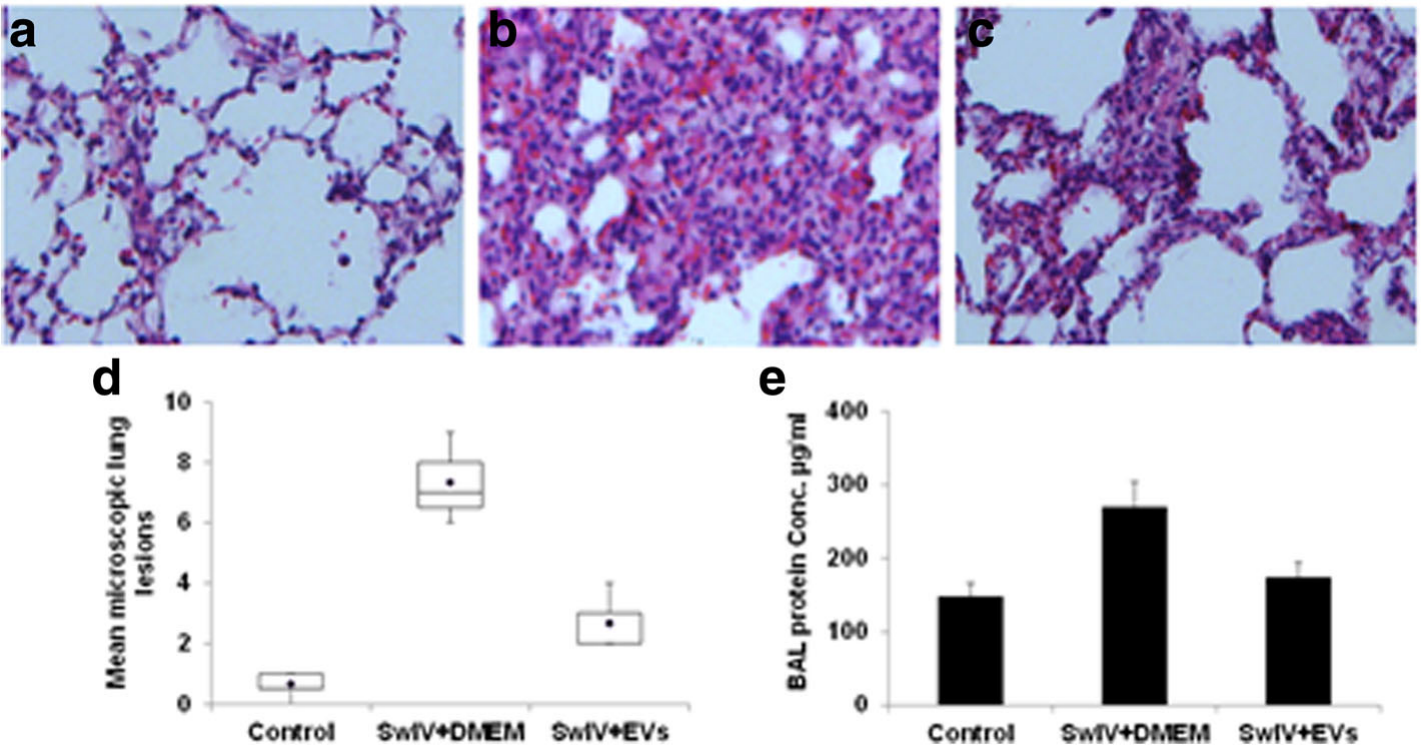
Effect of MSC-EV administration on microscopic lung lesions in pigs infected with SwIV. Eight-week-old pigs were mock infected or infected with swine/MN/08; H1N1 (SwIV). After 12 h, pigs were administered with Dulbecco’s modified Eagle’s medium (DMEM) or MSC extracellular vesicles (EVs). Three days after EV administration, pigs were euthanized and microscopic lung lesions and levels of total protein in bronchoalveolar lavage (BAL) were examined. **a** Control uninfected lung, showing normal alveolar walls, clear air space, and absence of exudation into the alveolar space. **b** SwIV induced exudative interstitial pneumonia characterized by thickened alveolar walls, collapsed alveolar spaces, and infiltration of inflammatory cells, whereas **c** lungs of pigs inoculated with MSC-EVs 12 h after SwIV infection show mild infiltration of inflammatory cells. Hematoxylin and eosin stain. Magnification × 200. **d,e** Histopathological scores. Lung tissue slides were examined for bronchiolar epithelial changes, peribronchiolar inflammation, and interstitial pneumonia, and lesions were scored from 0–3 (**d**). Values in each bar indicate mean microscopic lung lesions of three pigs ± SD. **e** MSC-EV administration decreased the levels of total protein in the BAL of SwIV + EV administered pigs as compared with SwIV + DMEM inoculated pigs. Data are expressed as mean levels of total protein in BAL of three pigs ± SD

SwIV induced extensive lung lesions in infected pigs as determined by infiltration of inflammatory cells, thickened alveolar walls, and collapsed alveoli, whereas lungs of pigs administered with MSC-EVs showed minor infiltration of inflammatory cells. The mean microscopic lung lesion score in SwIV + DMEM inoculated pigs was 7.3 ± 1.5 compared with 2.6 ± 1.5 in the SwIV + EV administered group (Fig. 7a–d). Consistent with the inflammatory lesions in lungs, the levels of total protein in the BAL of SwIV + EV (174 ± 20 μg/ml) administered pigs were also lower than the levels in SwIV + DMEM (270 ± 34 μg/ml) inoculated pigs (Fig. 7e).

**Fig. 8:**
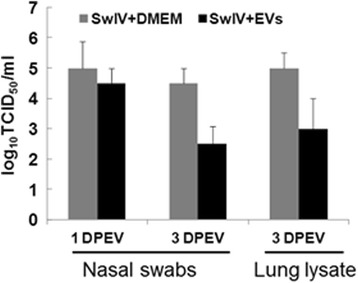
Effect of MSC-EV administration on virus titers in nasal swabs and lungs of pigs infected with SwIV. Eight-week-old pigs were mock infected or infected with swine/MN/08; H1N1 (SwIV). After 12 h, pigs were administered intratracheally with Dulbecco’s modified Eagle’s medium (DMEM) or MSC extracellular vesicles (EVs). Nasal swabs were collected from infected pigs at 1 and 3 days after EV administration (1DPEV and 3DPEV). At 3 days after EV administration, pigs were euthanized and lungs were harvested. Lung tissues were homogenized to prepare 10% lung lysate. Influenza virus shedding in nasal swabs and virus titers in lungs were determined by titration in MDCK cells. Values in each bar indicate mean virus titers of three pigs ± SD

We detected the influenza virus shedding in nasal swabs at 1 and 3 days after EV administration. Virus shedding was 100-fold lower in the EV-administered group at 3 days post-EV administration as compared with SwIV + DMEM inoculated pigs (Fig. 8). Similarly, virus titers were also 100-fold lower in the lung lysate of EV-administered pigs. These data suggest that MSC-EVs inhibit influenza virus replication and shedding in pigs.

### MSC-EVs attenuate SwIV-induced inflammatory cytokines

**Fig. 9:**
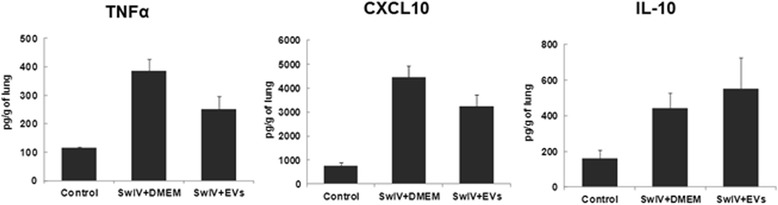
Effect of MSC-EV administration on cytokine production in lungs of SwIV-infected pigs. Eight-week-old pigs were mock infected or infected with swine/MN/08; H1N1 (SwIV). After 12 h, pigs were administered intratracheally with Dulbecco’s modified Eagle’s medium (DMEM) or MSC extracellular vesicles (EVs). Three days after EV administration, pigs were euthanized and cytokine production in lung lysate was analyzed by ELISA. Each bar represents mean concentrations of cytokines ± SD from three pigs. IL interleukin, TNF tumor necrosis factor

Next, we examined if MSC-EVs also modulated the inflammatory cytokine production in lungs. In the MSC-EV administered group, the levels of TNFα were 251 ± 46 pg/g lung lysate compared with 386 ± 40 pg/g in pigs inoculated with SwIV + DMEM. Similarly, levels of CXCL10 in SwIV + EV administered pigs were 3259 ± 469 pg/g compared with 4456 ± 495 pg/g in lung lysates of SwIV + DMEM inoculated pigs. The levels of the anti-inflammatory cytokine IL-10 were slightly higher in the SwIV + EV administered group compared with the SwIV + DMEM inoculated pigs (Fig. 9). These findings suggest that MSC-EVs possess anti-influenza and anti-inflammatory properties and attenuated influenza virus-induced ALI in a pig model.

## Discussion

In this study, we isolated porcine BM-MSC-derived EVs and examined their anti-viral and immunomodulatory properties in a pig model of influenza virus. MSC-EVs were round-shaped and, similarly to BM-MSCs, expressed mesenchymal markers but, unlike MSC, EVs lacked the expression of both SLA-I and II whereas MSCs expressed SLA-I. Importantly, MSC-EVs inhibited the HA activity of influenza viruses, suppressed the replication of influenza virus in lung epithelial cells, and inhibited influenza virus replication and proinflammatory cytokine production in the lungs of virus-infected pigs.

We isolated and characterized EVs from porcine BM-MSCs using protocols as described for human MSC-derived EVs [23, 29, 30]. As for human MSC-EVs, we also used TEM to determine the size and morphology of MSC-EVs. Size distribution of human MSC-EVs was also determined by nanotracking but this technique was not used in this study. Human MSC-derived EVs expressed mesenchymal markers and lacked the expression of MHC molecules [36]. In our study, pig MSC-derived EVs also expressed mesenchymal markers and the expression of both SLA-I and II was not detected in EVs, suggesting that pig MSC-EVs are similar to human MSC-EVs and will be useful in understanding the mechanisms of MSC-EV-mediated therapeutic effects in a pig model of human diseases.

A number of studies have demonstrated the beneficial effects of MSC-EVs in vitro and in animal models of lung, kidney, and liver diseases [37, 38]. Furthermore, several studies have shown that EVs exert their therapeutic effects by transferring their mRNA to diseased cells [20, 29, 39, 40]. In this study, the anti-influenza virus activity of MSC-EVs in lung epithelial cells was mediated by their mRNA content as pretreatment of EVs with RNase enzyme abrogated the anti-influenza virus activity of EVs. In a recent study, Qian and colleagues [41] demonstrated that exosomes (Exo) isolated from umbilical cord-derived MSCs inhibited replication of hepatitis C virus (HCV) in human dermal fibroblast 7 cells. These authors further identified four MSC-Exo-specific miRNAs with anti-HCV activity. MSC-EV-specific miRNAs have also been implicated in the regulation of cell survival, differentiation, and immunomodulation [42, 43]. Future studies will focus on identifying MSC-EV-specific mRNA and miRNAs that may play a role in regulating the anti-influenza activities of MSC-EVs.

One of the mechanisms by which influenza viruses cause extensive lung damage and mortality is due to the induction of unregulated inflammatory response [44, 45]. Similarly to MSCs, MSC-derived EVs also possess immunomodulatory activity [46–52]. In this study, MSC-EV inoculated pigs showed significantly less lung inflammation and decreased levels of proinflammatory cytokine and chemokine production. Additionally, increased levels of anti-inflammatory cytokine IL-10 were observed in MSC-EV administered pigs. These data suggest that, in addition to anti-influenza activity, MSC-EVs also exert their immunomodulatory activities and suppress the inflammatory response in influenza. Similar to our findings, MSC-EVs caused decreased production of inflammatory cytokines and increased production of IL-10 in a mouse model of ARDS [20]. Monsel and colleagues [23] demonstrated that MSC-EVs express cyclooxygenase (COX)-2 mRNA, the enzyme that induces prostaglandin E2 (PGE2) synthesis. PGE2 secreted by MSCs has been shown to reprogram proinflammatory monocyte-macrophages (M1) to the anti-inflammatory (M2) type that produces high levels of IL-10 [53]. Additionally, MSC-EVs interact with immune cells and cause the production of transforming growth factor (TGF)β and T-regulatory cells (Tregs) [48]. Tregs promote virus clearance and recovery in influenza virus-infected mice [54, 55]. In future studies, we will examine whether MSC-EV treatment induces the generation of M2-type macrophages and Tregs in a pig model of influenza virus.

MSCs mediate their immunomodulatory and tissue repair functions by the release of numerous therapeutic soluble factors [43, 56]. Indoleamine 2,3-dioxygenase (IDO) is produced by MSCs and modulates the functions of immune cells and inhibits influenza virus replication [57]. Similarly, LL37, an antimicrobial peptide, is produced by MSCs and has been shown to inhibit the growth of several species of bacteria and also inhibits replication of influenza virus [58, 59]. Additionally, LL37 was also shown to regulate the immunomodulatory activities of MSCs [60]. Currently, data on whether LL37 and IDO are transferred from MSCs to their secreted EVs are not available and were not examined in this study. It may be possible that these factors may be involved in mediating the anti-influenza and anti-inflammatory activities of MSC-EVs in our influenza model.

Recently, a few studies examined the therapeutic potential of MSCs in influenza in mice with conflicting results [9–12]. The Liles and Matthay groups reported that mouse and human MSCs failed to prevent influenza virus-induced mortality and ALI in mice [10, 11]. More recently, two publications have shown that MSCs reduced influenza virus-induced ALI in mice infected with HPAI H5N1 and H9N2 without affecting the virus replication [9, 12]. We and others have shown previously that influenza virus infected MSCs and that infected MSCs produced inflammatory cytokines [13–15]. Of the in vivo studies that examined the therapeutic efficacy of MSCs in influenza virus-induced ALI, none examined the infection of administered MSCs with influenza virus. Replicating virus in influenza virus-infected mice may infect the injected MSCs and virus-infected MSCs may not be functionally effective in inhibiting virus replication and inflammation. In in-vitro studies, influenza virus infection caused the apoptosis and lysis of infected MSCs [13]. Inactivated MSCs have been shown to retain some of their immunomodulatory functions. Heat-inactivated MSCs modulated the functions of monocytes/macrophages but had no effect on T or B cells [61]. In studies which demonstrated the protective effects of transplanted MSCs in influenza virus-infected mice, anti-inflammatory effects observed in these studies may likely be mediated by influenza-infected dead or apoptotic MSCs. Future experiments will be required to confirm the infection of transplanted MSC by influenza virus and to examine their interaction with immune cells in animal models of influenza virus.

MSC-EVs were recently identified and were as equally effective as MSCs in treating endotoxin and *E coli*-induced ALI in rodent models [20, 23]. Here, we demonstrated that MSC-EVs attenuated influenza virus-induced ALI in a pig model. Importantly, administration of MSC-EVs was safe since no gross pathological lesions were observed in any of the internal organs of the pigs. Additionally, MSC-EV treatment was also found to be safe in human patients treated for graft-versus-host disease (GvHD) and chronic kidney disease [50, 62]. Thus, the findings of this study may help in the planning of future clinical trials in humans using MSC-EVs as a cell-free therapy for ARDS.

MSC-EVs offer several advantages over MSCs as a therapy for human diseases. Although MSCs have been proven to be effective and safe in treating several human conditions in animal models and clinical trials, safety concerns such as pulmonary embolism, uncontrolled differentiation, and tumor formation are still associated with the use of MSCs as a therapy in humans [63, 64]. On the other hand, use of EVs as a therapy was found to be safer than MSCs [20, 50]. EVs are stable in circulation due to their lipid membrane, have no risk of aneuploidy, and are well tolerated in recipients due to their small size and lack of expression of MHC molecules. Moreover, EVs can be stored at –80 °C without losing their biochemical activity [65, 66]. Thus, EVs have the potential to become a safe and effective cell-free therapy for human diseases. However, a number of challenges, such as standard protocols for the production of clinical grade EVs, their dosing, quality control, and storage conditions, still need to be addressed before MSC-EVs can be advanced to the clinic.

This study has certain limitations: 1) an additional control group consisting of EVs derived from fibroblasts is needed to confirm that the beneficial effects in influenza are specifically mediated by MSC-EV; and 2) administration of EVs by the intravenous route would be more relevant in the clinical settings. However, in several studies, EVs obtained from MSCs, but not from fibroblasts, showed beneficial effects in ARDS when inoculated intravenously or intratracheally. In our preliminary studies in a pig model of lipopolysaccharide (LPS)-induced ARDS, only human MSC-derived EVs, and not EVs from normal human lung fibroblasts, attenuated lung inflammation.

## Conclusions

In summary, EVs derived from porcine BM-MSCs expressed mesenchymal markers, inhibited influenza virus replication in vitro and in vivo, and attenuated influenza virus-induced ALI in a relevant preclinical large animal model. Our findings suggest that MSC-EVs may be a potential cell-free therapy for acute inflammatory diseases including influenza.

## Abbreviations

ALI: Acute lung injury
ARDS: Acute respiratory distress syndrome
BAL: Bronchoalveolar lavage
BM: Bone marrow
BW: Body weight
CM: Conditioned medium
DAPI: 4′,6-Diamidino-2-phenylindole
DMEM: Dulbecco’s modified Eagle’s medium
DPI: Days post-infection
ELISA: Enzyme-linked immunosorbent assay
EV: Extracellular vesicle
Exo: Exosomes
FBS: Fetal bovine serum
HA: Hemagglutination
HCV: Hepatitis C virus
HPAI: Highly pathogenic avian influenza
IAV: Influenza A viruses
IDO: Indoleamine 2,3-dioxygenase
IFA: Immunofluorescence assay
IL: Interleukin
LEC: Lung epithelial cell
miRNA: MicroRNA
MV: Microvesicle
MSC: Mesenchymal stem (stromal) cell
NP: Nucleoprotein
PBS: Phosphate-buffered saline
PGE2: Prostaglandin E2
SLA: Swine leukocyte antigen
SwIV H1N1: Swine/MN/08
TEM: Transmission electron microscopy
TNF: Tumor necrosis factor
Treg: T regulatory cell

## References

[1] Beigel JH, Farrar J, Han AM, Hayden FG, Hyer R, de Jong MD (2005). Avian influenza A (H5N1) infection in humans. N Engl J Med..

[2] de Jong MD, Bach VC, Phan TQ, Vo MH, Tran TT, Nguyen BH (2005). Fatal avian influenza A (H5N1) in a child presenting with diarrhea followed by coma. N Engl J Med..

[3] de Jong MD, Simmons CP, Thanh TT, Hien VM, Smith GJ, Chau TN (2006). Fatal outcome of human influenza A (H5N1) is associated with high viral load and hypercytokinemia. Nat Med..

[4] Uccelli A, Moretta L, Pistoia V (2008). Mesenchymal stem cells in health and disease. Nat Rev Immunol..

[5] Laffey JG, Matthay MA (2017). Fifty years of research in ARDS. Cell-based therapy for acute respiratory distress syndrome. biology and potential therapeutic value. Am J Respir Crit Care Med.

[6] Wilson JG, Liu KD, Zhuo H, Caballero L, McMillan M, Fang X (2015). Mesenchymal stem (stromal) cells for treatment of ARDS: a phase 1 clinical trial. Lancet Respir Med..

[7] Zheng G, Huang L, Tong H, Shu Q, Hu Y, Ge M (2014). Treatment of acute respiratory distress syndrome with allogeneic adipose-derived mesenchymal stem cells: a randomized, placebo-controlled pilot study. Respir Res..

[8] Khatri M, Dwivedi V, Krakowka S, Manickam C, Ali A, Wang L (2010). Swine influenza H1N1 virus induces acute inflammatory immune responses in pig lungs: a potential animal model for human H1N1 influenza virus. J Virol..

[9] Chan MC, Kuok DI, Leung CY, Hui KP, Valkenburg SA, Lau EH (2016). Human mesenchymal stromal cells reduce influenza A H5N1-associated acute lung injury in vitro and in vivo. Proc Natl Acad Sci U S A..

[10] Darwish I, Banner D, Mubareka S, Kim H, Besla R, Kelvin DJ (2013). Mesenchymal stromal (stem) cell therapy fails to improve outcomes in experimental severe influenza. PLoS One..

[11] Gotts JE, Abbott J, Matthay MA (2014). Influenza causes prolonged disruption of the alveolar-capillary barrier in mice unresponsive to mesenchymal stem cell therapy. Am J Physiol Lung Cell Mol Physiol..

[12] Li Y, Xu J, Shi W, Chen C, Shao Y, Zhu L (2016). Mesenchymal stromal cell treatment prevents H9N2 avian influenza virus-induced acute lung injury in mice. Stem Cell Res Ther..

[13] Khatri M, O'Brien TD, Goyal SM, Sharma JM (2010). Isolation and characterization of chicken lung mesenchymal stromal cells and their susceptibility to avian influenza virus. Dev Comp Immunol..

[14] Khatri M, Saif YM (2013). Influenza virus infects bone marrow mesenchymal stromal cells in vitro: implications for bone marrow transplantation. Cell Transplant..

[15] Thanunchai M, Kanrai P, Wiboon-Ut S, Puthavathana P, Hongeng S, Thitithanyanont A (2013). Tropism of avian influenza A (H5N1) virus to mesenchymal stem cells and CD34+ hematopoietic stem cells. PLoS One..

[16] Phinney DG, Prockop DJ (2007). Concise review: mesenchymal stem/multipotent stromal cells: the state of transdifferentiation and modes of tissue repair—current views. Stem Cells..

[17] Yeo RW, Lai RC, Zhang B, Tan SS, Yin Y, Teh BJ (2013). Mesenchymal stem cell: an efficient mass producer of exosomes for drug delivery. Adv Drug Deliv Rev..

[18] Kim HS, Choi DY, Yun SJ, Choi SM, Kang JW, Jung JW (2012). Proteomic analysis of microvesicles derived from human mesenchymal stem cells. J Proteome Res..

[19] Phinney DG, Di Giuseppe M, Njah J, Sala E, Shiva S, St Croix CM, et al. Mesenchymal stem cells use extracellular vesicles to outsource mitophagy and shuttle microRNAs. Nat Commun. 2015;6:8472. 10.1038/ncomms9472 PMC4598952 26442449

[20] Zhu YG, Feng XM, Abbott J, Fang XH, Hao Q, Monsel A (2014). Human mesenchymal stem cell microvesicles for treatment of Escherichia coli endotoxin-induced acute lung injury in mice. Stem Cells..

[21] Camussi G, Deregibus MC, Cantaluppi V (2013). Role of stem cell-derived microvesicles in the paracrine action of stem cells. Biochem Soc Trans..

[22] Quesenberry PJ, Aliotta JM (2010). Cellular phenotype switching and microvesicles. Adv Drug Deliv Rev..

[23] Monsel A, Zhu YG, Gennai S, Hao Q, Hu S, Rouby JJ (2015). Therapeutic effects of human mesenchymal stem cell-derived microvesicles in severe pneumonia in mice. Am J Respir Crit Care Med..

[24] Rogers CS, Abraham WM, Brogden KA, Engelhardt JF, Fisher JT, McCray PB (2008). The porcine lung as a potential model for cystic fibrosis. Am J Physiol Lung Cell Mol Physiol..

[25] Walters EM, Wells KD, Bryda EC, Schommer S, Prather RS (2017). Swine models, genomic tools and services to enhance our understanding of human health and diseases. Lab Anim (NY).

[26] Kuiken T, Taubenberger JK (2008). Pathology of human influenza revisited. Vaccine..

[27] Khatri M, O'Brien TD, Sharma JM (2009). Isolation and differentiation of chicken mesenchymal stem cells from bone marrow. Stem Cells Dev..

[28] Pittenger MF, Mackay AM, Beck SC, Jaiswal RK, Douglas R, Mosca JD (1999). Multilineage potential of adult human mesenchymal stem cells. Science..

[29] Bruno S, Grange C, Deregibus MC, Calogero RA, Saviozzi S, Collino F (2009). Mesenchymal stem cell-derived microvesicles protect against acute tubular injury. J Am Soc Nephrol..

[30] Cruz FF, Borg ZD, Goodwin M, Sokocevic D, Wagner DE, Coffey A (2015). Systemic administration of human bone marrow-derived mesenchymal stromal cell extracellular vesicles ameliorates aspergillus hyphal extract-induced allergic airway inflammation in immunocompetent mice. Stem Cells Transl Med..

[31] Thery C, Boussac M, Veron P, Ricciardi-Castagnoli P, Raposo G, Garin J, Amigorena S (2001). Proteomic analysis of dendritic cell-derived exosomes: a secreted subcellular compartment distinct from apoptotic vesicles. J Immunol..

[32] Khatri M, O'Brien TD, Chattha KS, Saif LJ (2015). Porcine lung mesenchymal stromal cells possess differentiation and immunoregulatory properties. Stem Cell Res Ther..

[33] Thomas M, Wang Z, Sreenivasan CC, Hause BM, Gourapura JR, Li F (2015). Poly I:C adjuvanted inactivated swine influenza vaccine induces heterologous protective immunity in pigs. Vaccine..

[34] Richt JA, Lager KM, Janke BH, Woods RD, Webster RG, Webby RJ (2003). Pathogenic and antigenic properties of phylogenetically distinct reassortant H3N2 swine influenza viruses cocirculating in the United States. J Clin Microbiol..

[35] Jung K, Renukaradhya GJ, Alekseev KP, Fang Y, Tang Y, Saif LJ (2009). Porcine reproductive and respiratory syndrome virus modifies innate immunity and alters disease outcome in pigs subsequently infected with porcine respiratory coronavirus: implications for respiratory viral co-infections. J Gen Virol..

[36] De Luca L, Trino S, Laurenzana I, Simeon V, Calice G, Raimondo S (2016). MiRNAs and piRNAs from bone marrow mesenchymal stem cell extracellular vesicles induce cell survival and inhibit cell differentiation of cord blood hematopoietic stem cells: a new insight in transplantation. Oncotarget..

[37] Monsel A, Zhu YG, Gudapati V, Lim H, Lee JW (2016). Mesenchymal stem cell derived secretome and extracellular vesicles for acute lung injury and other inflammatory lung diseases. Expert Opin Biol Ther..

[38] Rani S, Ryan AE, Griffin MD, Ritter T (2015). Mesenchymal stem cell-derived extracellular vesicles: toward cell-free therapeutic applications. Mol Ther..

[39] Ragni E, Banfi F, Barilani M, Cherubini A, Parazzi V, Larghi P (2017). Extracellular vesicle-shuttled mRNA in mesenchymal stem cell communication. Stem Cells..

[40] Tomasoni S, Longaretti L, Rota C, Morigi M, Conti S, Gotti E (2013). Transfer of growth factor receptor mRNA via exosomes unravels the regenerative effect of mesenchymal stem cells. Stem Cells Dev..

[41] Qian X, Xu C, Fang S, Zhao P, Wang Y, Liu H (2016). Exosomal microRNAs derived from umbilical mesenchymal stem cells inhibit hepatitis C virus infection. Stem Cells Transl Med..

[42] Baglio SR, Pegtel DM, Baldini N (2012). Mesenchymal stem cell secreted vesicles provide novel opportunities in (stem) cell-free therapy. Front Physiol..

[43] Bruno S, Deregibus MC, Camussi G (2015). The secretome of mesenchymal stromal cells: role of extracellular vesicles in immunomodulation. Immunol Lett..

[44] Herold S, Becker C, Ridge KM, Budinger GR (2015). Influenza virus-induced lung injury: pathogenesis and implications for treatment. Eur Respir J..

[45] Short KR, Kroeze EJ, Fouchier RA, Kuiken T (2014). Pathogenesis of influenza-induced acute respiratory distress syndrome. Lancet Infect Dis..

[46] Blazquez R, Sanchez-Margallo FM, de la Rosa O, Dalemans W, Alvarez V, Tarazona R (2014). Immunomodulatory potential of human adipose mesenchymal stem cells derived exosomes on in vitro stimulated T cells. Front Immunol..

[47] Budoni M, Fierabracci A, Luciano R, Petrini S, Di Ciommo V, Muraca M (2013). The immunosuppressive effect of mesenchymal stromal cells on B lymphocytes is mediated by membrane vesicles. Cell Transplant..

[48] Chen W, Huang Y, Han J, Yu L, Li Y, Lu Z (2016). Immunomodulatory effects of mesenchymal stromal cells-derived exosome. Immunol Res..

[49] Favaro E, Carpanetto A, Lamorte S, Fusco A, Caorsi C, Deregibus MC (2014). Human mesenchymal stem cell-derived microvesicles modulate T cell response to islet antigen glutamic acid decarboxylase in patients with type 1 diabetes. Diabetologia..

[50] Kordelas L, Rebmann V, Ludwig AK, Radtke S, Ruesing J, Doeppner TR (2014). MSC-derived exosomes: a novel tool to treat therapy-refractory graft-versus-host disease. Leukemia..

[51] Mokarizadeh A, Delirezh N, Morshedi A, Mosayebi G, Farshid AA, Mardani K (2012). Microvesicles derived from mesenchymal stem cells: potent organelles for induction of tolerogenic signaling. Immunol Lett..

[52] Zhang B, Yin Y, Lai RC, Tan SS, Choo AB, Lim SK (2014). Mesenchymal stem cells secrete immunologically active exosomes. Stem Cells Dev..

[53] Nemeth K, Leelahavanichkul A, Yuen PS, Mayer B, Parmelee A, Doi K (2009). Bone marrow stromal cells attenuate sepsis via prostaglandin E(2)-dependent reprogramming of host macrophages to increase their interleukin-10 production. Nat Med..

[54] Moser EK, Hufford MM, Braciale TJ (2014). Late engagement of CD86 after influenza virus clearance promotes recovery in a FoxP3+ regulatory T cell dependent manner. PLoS Pathog..

[55] Oliphant S, Lines JL, Hollifield ML, Garvy BA (2015). Regulatory T cells are critical for clearing influenza A virus in neonatal mice. Viral Immunol..

[56] Gallina C, Turinetto V, Giachino C (2015). A new paradigm in cardiac regeneration: the mesenchymal stem cell secretome. Stem Cells Int..

[57] Li F, Karlsson H (2017). Antiviral effect of IDO in mouse fibroblast cells during influenza virus infection. Viral Immunol..

[58] Krasnodembskaya A, Song Y, Fang X, Gupta N, Serikov V, Lee JW (2010). Antibacterial effect of human mesenchymal stem cells is mediated in part from secretion of the antimicrobial peptide LL-37. Stem Cells..

[59] Tripathi S, Tecle T, Verma A, Crouch E, White M, Hartshorn KL (2013). The human cathelicidin LL-37 inhibits influenza A viruses through a mechanism distinct from that of surfactant protein D or defensins. J Gen Virol..

[60] Oliveira-Bravo M, Sangiorgi BB, Schiavinato JL, Carvalho JL, Covas DT, Panepucci RA (2016). LL-37 boosts immunosuppressive function of placenta-derived mesenchymal stromal cells. Stem Cell Res Ther..

[61] Luk F, de Witte SF, Korevaar SS, Roemeling-van Rhijn M, Franquesa M, Strini T (2016). Inactivated mesenchymal stem cells maintain immunomodulatory capacity. Stem Cells Dev..

[62] Nassar W, El-Ansary M, Sabry D, Mostafa MA, Fayad T, Kotb E (2016). Umbilical cord mesenchymal stem cells derived extracellular vesicles can safely ameliorate the progression of chronic kidney diseases. Biomater Res..

[63] Haarer J, Johnson CL, Soeder Y, Dahlke MH (2015). Caveats of mesenchymal stem cell therapy in solid organ transplantation. Transpl Int..

[64] Heslop JA, Hammond TG, Santeramo I, Tort Piella A, Hopp I, Zhou J (2015). Concise review: workshop review: understanding and assessing the risks of stem cell-based therapies. Stem Cells Transl Med..

[65] Kalimuthu S, Gangadaran P, Li XJ, Oh JM, Lee HW, Jeong SY (2016). In vivo therapeutic potential of mesenchymal stem cell-derived extracellular vesicles with optical imaging reporter in tumor mice model. Sci Rep..

[66] Reis M, Ogonek J, Qesari M, Borges NM, Nicholson L, Preussner L (2016). Recent developments in cellular immunotherapy for HSCT-associated complications. Front Immunol..

